# Starch–ZnAl Layered Double-Hydroxide Nanocomposites and PVDF Membrane Nanofillers for the Sustainable Recovery of Dye-Contaminated Water

**DOI:** 10.3390/polym18182248

**Published:** 2026-09-15

**Authors:** Mukarram Zubair, Nuhu Dalhat Muazu, Taye Saheed Kazeem, Muhammad Daud, Mohammad Saood Manzar, Hamza Zahir, Hessa Al-Qahtani, Ahmad Hussaini Jagaba, Omer Aga, Jwaher M. AlGhamdi, Munirah Abdullah Al-Messiere

**Affiliations:** 1 Environmental Engineering Department, College of Engineering A13, Imam Abdulrahman Bin Faisal University, Main Campus, P.O. Box 1982, Dammam 34212, Saudi Arabia; msmanzar@iau.edu.sa (M.S.M.); oaga@iau.edu.sa (O.A.); 2 School of Chemical Engineering, The University of New South Wales, Sydney, NSW 2052, Australia; t.kazeem@unswalumni.com; 3 Department of Chemical Engineering, Faculty of Mechanical, Chemical and Industrial Engineering, University of Engineering and Technology, Peshawar 25120, Pakistan; hamza.zaahirr@gmail.com; 4 Interdisciplinary Research Center for Refining & Advanced Chemicals (IRC-RAC) Research Institute, King Fahd University of Petroleum & Minerals, Dhahran 31261, Saudi Arabia; 5 Department of Chemistry, College of Science, Imam Abdulrahman Bin Faisal University, P.O. Box 1982, Dammam 31441, Saudi Arabia; hlqahtani@iau.edu.sa (H.A.-Q.); jmalghamdi@iau.edu.sa (J.M.A.); 6 Department of Civil Engineering, Faculty of Engineering, University of Tabuk, P.O. Box 741, Tabuk 71491, Saudi Arabia; ajagaba@ut.edu.sa; 7 Department of Physics, College of Science, Imam Abdulrahman Bin Faisal University, P.O. Box 1982, Dammam 31441, Saudi Arabia; malmessiere@iau.edu.sa

**Keywords:** bio-based nanocomposites, circular water management, mixed-matrix poly(vinylidene fluoride) membranes, polymeric nanocomposites membranes, bio-based nanofillers, LDH nanocomposites, membrane separation purification, regeneration and reuse water resource recovery

## Abstract

This study presents a starch-modified calcined-ZnAl layered double-hydroxide (S-C-ZnAl-LDH) nanocomposite as a multifunctional nanofiller for poly(vinylidene fluoride) (PVDF) efficiently performing, simultaneously, ultrafiltration membrane filtration and efficient adsorbent for the recovery of Acid Blue dye-contaminated water. The synergistic effects of starch modification and thermal activation on nanofiller structure, interfacial compatibility, and membrane performance were systematically investigated through a comparison with pristine ZnAl-LDH, calcined ZnAl-LDH, starch-modified ZnAl-LDH, and calcined starch-modified ZnAl-LDH. SEM, TEM, and XRD analyses confirmed the formation of hierarchical layered nanosheet architectures with a uniform dispersion of crystalline ZnAl domains within a partially amorphous starch matrix, promoting enhanced polymer–nanofiller interfacial interactions Adsorption performance was influenced by solution pH, initial dye concentration, and temperature. Nonlinear kinetic analysis showed that the PFO model described the kinetic data better. However, the overall kinetic modeling findings suggest that Acid Blue 92 adsorption is governed by a combination of physicochemical interactions, suggesting a complex adsorption mechanism was involved. The starch-modified nanocomposite exhibited excellent regeneration stability, retaining approximately 88–90% of its adsorption capacity after five adsorption–desorption cycles. More importantly, the incorporation of S-C-ZnAl-LDH into PVDF membranes significantly enhanced membrane functionality, increasing water flux and permeance by 42.9% and 25%, respectively, while improving Acid Blue rejection by 35.7% to approximately 98%. These improvements are attributed to enhanced membrane hydrophilicity, optimized nanofiller dispersion, and favorable polymer–filler interfacial interactions that facilitate water transport while maintaining high separation efficiency. This work demonstrates an effective strategy for integrating renewable bio-based modifiers with layered nanomaterials to engineer advanced polymeric films exhibiting enhanced permeability, selectivity, durability, and reusability, providing a sustainable platform for multifunctional membrane technologies in water purification and environmental protection.

## 1. Introduction

Water plays a crucial role in our daily lives, yet it faces significant pollution challenges from various sources. Key contributors to water pollution include industrial, domestic, and agricultural activities, alongside other environmental and global changes [1,2]. In many regions of the world, several instances of surface and groundwater contamination have been reported, rendering the water unsafe for drinking. With the current global population and urban development growth trend, the increasing water demand has been projected to result in a severe shortage of clean potable and non-potable water in the future [1]. Adding to this concern, rapid industrialization in modern societies has significantly increased water pollution, particularly through the discharge of a wide range of industrial contaminants, such as synthetic dyes, into aquatic environments [2,3], which are widely used in several chemical industries, such as textiles, leather, paper, and cosmetics [4,5]. These dyes are often toxic, resistant to degradation, and present serious environmental and health hazards. When discharged inappropriately, these dyes have a direct impact on the water supply and can eventually reach living organisms such as the skin, lungs, and digestive systems, making them susceptible to several diseases because of their mutagenic and carcinogenic properties [6]. Additionally, dye pollutants in water pose significant environmental problems, including reduced light penetration, which disrupts aquatic ecosystems, and toxicity, which can harm both aquatic organisms and humans [7]. Among these dyes, Acid Blue, commonly employed in textile dying, are highly present in water. Their complex aromatic molecular structures make them highly resistant to natural degradation, posing a significant challenge for aqueous-medium remediation. While traditional dye removal methods, such as coagulation and flocculation [8], chemical oxidation [8], and biological treatment [9], can be somewhat effective, they often have notable drawbacks, including high operational costs, the generation of secondary pollutants, and limited effectiveness in treating large volumes of wastewater with low dye concentrations. Consequently, there is a growing demand for innovative, cost-efficient, and environmentally sustainable technologies for dye removal.

Adsorption has gained recognition as one of the most effective techniques for dye removal from wastewater due to its simplicity, affordability, high efficiency, and ease of operation [10]. Common types of adsorbents include activated carbon [11], biochar [12], and layered double hydroxides (LDHs) [13]. Each of these materials offers unique advantages; for instance, activated carbon provides high porosity and adsorption capacity. In this regard, layered double hydroxides (LDHs), also referred to as anionic clays, have garnered considerable attention as adsorbents. LDHs have a unique layered structure where positively charged metal hydroxide layers are interspersed with charge-balance anions in the interlayer spaces. This structure provides LDHs with a large surface area, strong anion-exchange capacity, and excellent adsorption properties, making them highly suitable for removing anionic dyes from water [14,15].

Starch, recognized for its high biodegradability, non-toxic nature, cost-effectiveness, eco-friendliness, and functional structure, is the most abundant, naturally occurring biopolymer widely used in various applications [16,17]. The presence of numerous functional groups in starch molecules can increase or activate the number of available sites for adsorption. The composite structure formed by combining starch and LDHs is expected to enhance both physical and chemical properties, resulting in improved adsorption capabilities due to the synergistic effects of their components.

Recent research has investigated the application of biopolymer-modified LDHs, including sodium alginate, starch, and chitosan-based composites, to improve adsorption efficiency; however, more detailed studies are needed on the specific combination of starch and ZnAl-LDHs for dye removal [18,19,20]. Starch–ZnAl layered double hydroxides (starch–ZnAl-LDH) are a specialized type of LDH, consisting of zinc and aluminum cations arranged in brucite-like layers, with starch serving as a functional modifier. Starch, a renewable and biodegradable nature polymer, is incorporated to enhance both the adsorption performance and biocompatibility of LDHs. The positive charge of starch–ZnAl-LDH layers is neutralized by anions, which can be exchanged with other anionic species in the solution. This feature makes starch–ZnAl-LDH particularly effective for adsorbing anionic pollutants, like Acid Blue dyes, through mechanisms such as ion-exchange, electrostatic attraction, and increased adsorption capacity, providing advantages such as environmental sustainability, cost-effectiveness, and the easy availability of raw materials. It is essential to understand the adsorption kinetics, isotherms, and thermodynamics, as well as the impact of factors such as pH, temperature, and initial dye concentration, to optimize the performance of starch–ZnAl-LDH-based adsorbents. This study aims, for the first time in the literature, to explore the potential of the incorporation of starch into ZnAl-LDHs (calcined and uncalcined) and assess its performance for the remediation of dye-laden water. Accordingly, the research focused on synthesizing starch–ZnAl-LDH with and without calcination, tested it for removing Acid Blue dye from an aqueous medium, and characterized its structural and surface properties using techniques such as X-ray diffraction (XRD), Fourier-transform infrared spectroscopy (FTIR), and scanning electron microscopy (SEM). Additionally, batch adsorption experiments were conducted to evaluate the effects of various parameters, including pH, contact time, adsorbent dosage, and initial dye concentration, on the adsorption capacity of starch–ZnAl-LDHs. Adsorption kinetics and isotherm models were created to analyze the data and gain insights into the adsorption mechanism.

Although layered double hydroxides have been extensively explored as adsorbents and polymer nanofillers, no previous study has systematically investigated the combined effects of renewable starch modification and calcination on the structure–property–performance relationships governing both adsorption and PVDF membrane functionality within a unified material platform. This work introduces a novel starch–ZnAl-LDH nanocomposite that simultaneously serves as a recyclable adsorbent and a multifunctional membrane nanofiller. It is expected that the presence of hydroxyl groups in starch coupled with ZnAl LDH not only enhances active binding sites for dye-contaminant remediation but also improves the dispersion of the ZnAl nanofiller within the PVDF matrix, which facilitates dye rejection. Through comprehensive characterization and performance evaluation, this study establishes how bio-based interfacial engineering and thermal activation enhance nanofiller dispersion, polymer–filler compatibility, membrane permeability, dye rejection, and regeneration stability. This approach provides new insights into the synthesis of sustainable polymer nanocomposite membranes for multifunctional water purification and environmental applications.

## 2. Materials and Methods

### 2.1. Materials

Aluminum(III) nitrate nonahydrate [Al(NO_3_)_3_·9H_2_O], zinc(II) nitrate hexahydrate [Zn(NO_3_)_2_·6H_2_O], soluble starch, sodium hydroxide (NaOH), nitric acid (HNO_3_), Acid Blue 92 dye, polyvinylidene fluoride (PVDF) pellets, N,N-dimethylformamide (DMF), and all other analytical-grade chemicals and reagents were purchased from Sigma-Aldrich (St. Louis, MO, USA) and used without further purification. Deionized (DI) water was used throughout the synthesis, adsorption, regeneration, membrane fabrication, and filtration experiments.

### 2.2. Synthesis of ZnAl Layered Double Hydroxides (ZnAl-LDHs) and Starch Composites

ZnAl-LDH nanocomposites were synthesized through the co-precipitation technique by dissolving zinc nitrate and aluminum nitrate in deionized water at a 2:1 molar ratio. This solution was slowly added dropwise to a sodium hydroxide solution, ensuring the pH was maintained at 10. The mixture was stirred continuously for 24 h, after which the precipitate was filtered, thoroughly washed with water, and dried at 80 °C to yield ZnAl-LDH powder. In a separate solution, 1 g of starch was dissolved under mild heating (approximately 60 °C). The metal nitrate solution was then gradually added to the starch solution while maintaining the pH at 10 using sodium hydroxide. After 24 h of continuous stirring, the resulting precipitate was filtered, washed, and dried at 80 °C to produce starch–ZnAl-LDH nanocomposites.

### 2.3. Characterization of Starch–ZnAl LDH Nanocomposites

The structural, morphological, and thermal characteristics of the synthesized starch–ZnAl layered double hydroxide (starch–ZnAl LDH) nanocomposites were comprehensively evaluated. Crystal structure and phase identification were determined via X-ray diffraction (XRD, Rigaku, Japan) using Cu Kα radiation (λ = 1.5428 Å) over a 2θ range of 5–90° at room temperature. Functional groups on the nanocomposite surfaces were identified using Fourier-transform infrared spectroscopy (FTIR, Nicolet 6700) across a range of 400–4000 cm^−1^ at a spectral resolution of 4 cm^−1^. Surface morphology and elemental distribution were examined using scanning electron microscopy (SEM, JSM-IT100, Japan) operated at an accelerating voltage in the range of 5–30 kV, while internal microstructural features were analyzed via transmission electron microscopy (TEM, JEM-2100, Japan). The hydrophilicity of ZnAl LDH and starch–ZnAl LDH membranes were performed using an Ossila contact-angle geniometer instrument, The Netherland.

### 2.4. RSM Methodology

This study employed the response surface methodology under the central composite design (CCD) to understand the effects of the combination of the adsorption-process operational parameters that included initial Acid Blue dye concentration in solution, pH, and temperature on the removal of Acid Blue dye from the aqueous phase. Accordingly, the three-level three-factor CCD adopted required a total of 17 experiments, which were performed in duplicates. The low, middle, and high levels of the independent variables were coded as −1, 0, and +1, respectively. With the aid of Design of Expert 11 software, the influence as well as the statistical significance of the independent variables on the dependent variable were evaluated using analysis of variance (ANOVA) based on the empirical RSM quadratic models we developed. Table 1 presents the selected ranges of independent parameters and their coded values.

### 2.5. Kinetic Modeling

The adsorption kinetics of Acid Blue 92 in ZnAl-LDH, ZnAl-LDH-C, ZnAl-LDH-S, and ZnAl-LDH-CS were evaluated using the pseudo-first-order model originally associated with the Lagergren kinetic formulation [21] and the pseudo-second-order model described by Ho and McKay [22]. The nonlinear form of the pseudo-first-order model is:

(1) $$q_{t}=q_{e}\left(1-e^{-k_{1}t}\right)$$

The nonlinear form of the pseudo-second-order model is:

(2) $$q_{t}=\frac{k_{2}q_{e}^{2}t}{1+k_{2}q_{e}t}$$

where $q_{t}$ and $q_{e}$ are the adsorption capacities at time t and equilibrium, respectively, expressed in mg g^−1^; t is the contact time in min^−1^; $k_{1}$ is the pseudo-first-order rate constant in min; and $k_{2}\;$is the pseudo-second-order rate constant in g mg^−1^ min^−1^. The initial adsorption rate was calculated as $h=k_{2}q_{e}^{2}$.

Model parameters were estimated by nonlinear least-squares regression using the original $q_{t}$ versus t data. Model performance was evaluated using the coefficient of determination (R^2^), root mean square error (RMSE), agreement between experimental and calculated equilibrium capacities, and residual analysis.

### 2.6. Regeneration

Regeneration experiments were conducted to evaluate the reusability of the starch–LDH adsorbent. A sorption–desorption cycle was performed using 100 mL of dye solution containing 50 mg of the adsorbent, agitated at 272 rpm for 6 h. After adsorption, the spent adsorbent was treated in 50 mL of 1 M NaOH solution and stirred for 2 h to facilitate dye desorption. The regenerated adsorbent was then centrifuged, thoroughly rinsed with deionized water, and dried at 60 °C for a range of 3–4 h. This regenerated material was reused for subsequent adsorption cycles. The adsorption–regeneration process was repeated for three additional cycles to assess the material’s stability and performance over multiple uses.

### 2.7. Synthesis of PVDF–Starch–LDH Membranes

The synthesis of pure PVDF, PVDF-LDH, and the PVDF–starch–LDH composite membrane was performed using the phase inversion method. Initially, 15% of PVDF pellets was dissolved in DMF solvent and agitated overnight at 40 °C to obtain a homogeneous solution. Similarly, the composite membranes were fabricated by taking 0.01 g of LDH or starch–LDH composite and ultrasonicated for 10 min (60 Hz) in DMF solvent. The dispersed composite was mixed with 15% PVDF pellets and agitated for 24 h at 40 °C. The mixture was degassed for 60 min and then dispersed on a glass plate using a doctor blade set at a thickness of 200 µm. The cast solution was transferred to a coagulation bath containing double-distilled water and kept for 24 h to finalize the formation of a membrane sheet. After the removal of the formed membrane from the bath, the prepared membranes were allowed to dry for 24 h and were then stored in a sealed pack to be ready for the filtration test.

### 2.8. Filtration Experiment

Membrane testing was performed by the dead-end filtration method using a Sterlitech HP4750 stirred cell. Initially, the prepared membranes were soaked in DI water for one hour. The concentration of Acid Blue 92 dye used was 80 ppm at a solution pH of 2, a fixed applied pressure of 2 bar, and a volume of 40 mL. The filtrate Acid Blue dye concentration was measured by a DR Hach 6000 UV-Vis spectrophotometer, USA at an analytical wavelength (λmax) of 570 nm.

## 3. Results

### 3.1. Characterization of ZnAl and Starch–ZnAl Composites

#### 3.1.1. SEM Analysis

**Figure 1:**
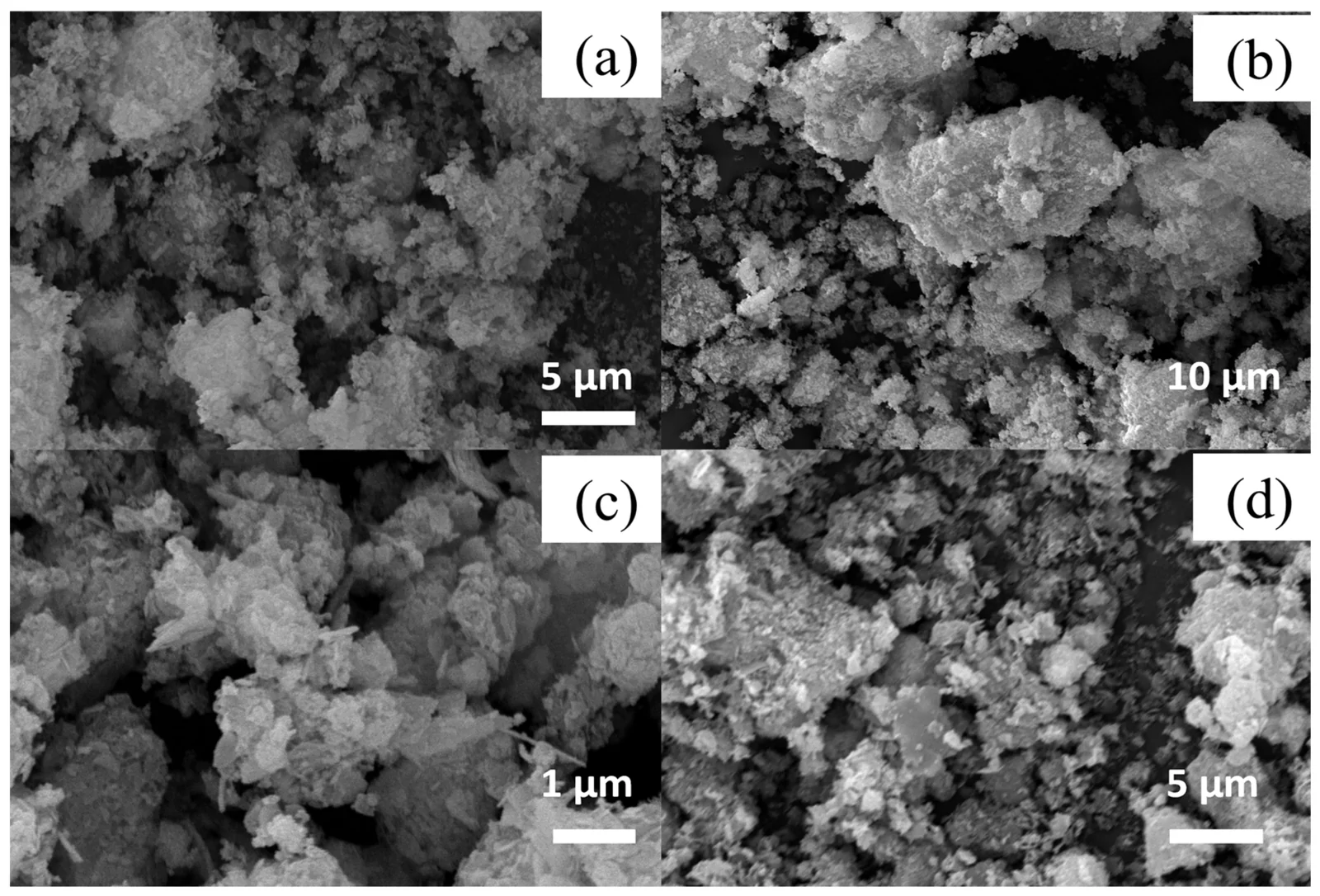
SEM images of (**a**) ZnAl-LDH; (**b**) calcined ZnAl-LDH; (**c**) starch–ZnAl; (**d**) calcined starch–ZnAl-LDH.

The morphological characteristics and surface properties of ZnAl-LDH and its composites were examined under scanning electron microscopy at various resolutions. Figure 1a reveals ZnAl-LDH agglomerated particles with irregular shapes and a rough surface texture. These particles are densely packed and primarily fall within the micron-size range, indicating the successful formation of LDHs. This morphology suggests a large surface area, making it well-suited for wastewater treatment applications. The rough surface morphology and interconnected particle arrangement can provide additional adsorption sites by increasing surface accessibility, which is beneficial for the interaction between pollutants and active hydroxyl groups of LDH materials [23]. Figure 1b reveals calcined ZnAl-LDH agglomerated particles with a rough, porous texture, suggesting structural changes caused by the removal of water and hydrogen layers during the calcination process. This morphology is characteristic of materials subjected to thermal treatment, leading to an increased surface area, which improves their catalytic properties. The formation of porous features after calcination is associated with the elimination of interlayer water and hydroxyl groups, resulting in structural rearrangement and the generation of mixed metal-oxide phases with enhanced surface activity [24]. Figure 1c shows ZnAl–starch aggregated particles with irregular shapes, indicating strong interparticle interactions. The presence of starch likely acts as a binder, enhancing the structural integrity and compactness of the composite. The starch polymer matrix can provide a support network around ZnAl-LDH particles, improving particle stabilization and limiting excessive aggregation through interactions between hydroxyl groups of starch and surface metal hydroxyl species [20,25,26]. This morphology suggests the effective incorporation of starch into the LDH. Figure 1d reveals calcined ZnAl–starch nanocomposites with a porous and fragmented structure with smaller, irregular particles. The calcination process seems to have caused structural collapse, resulting in a more disordered morphology, which likely contributed to an increased surface area and improved reactivity [27,28]. The fragmented morphology generated during calcination can expose more active sites and create diffusion pathways, which may enhance the accessibility of adsorption sites during wastewater treatment applications [29].

#### 3.1.2. TEM Analysis

**Figure 2:**
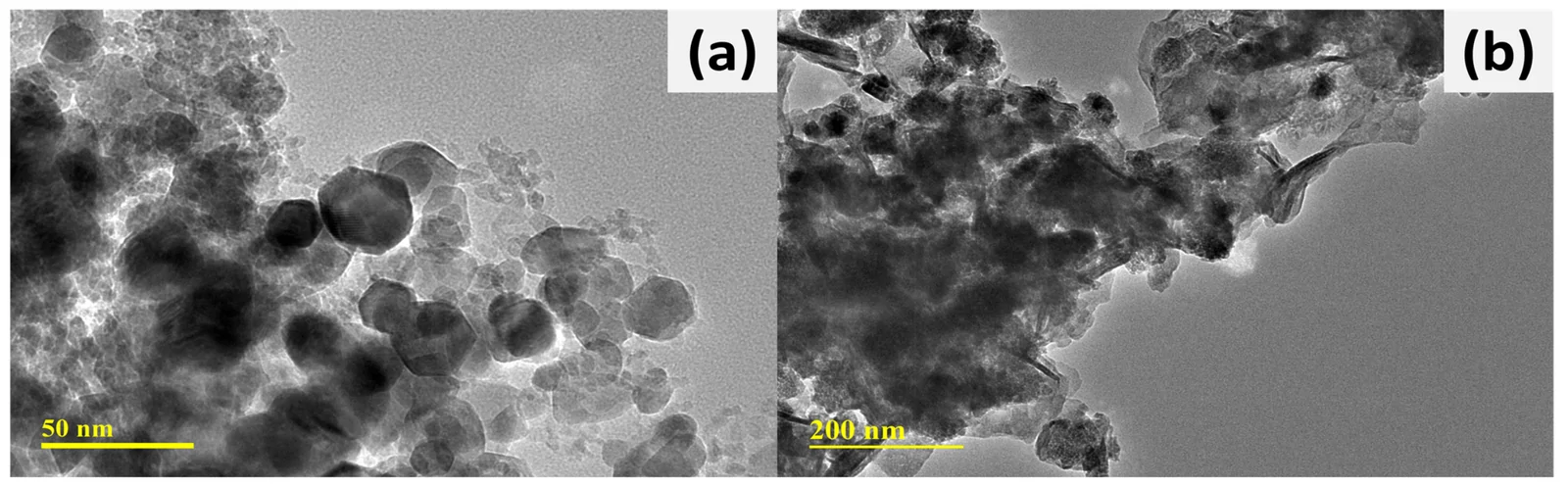
TEM images of (**a**) ZnAl-LDH and (**b**) starch–ZnAl-LDH.

The structural morphology of the fabricated adsorbents was examined through transmission electron microscopy (TEM), as depicted in Figure 2. The detailed topological micrographs of individual nanocomposites assisted us in examining the nanoscale features of adsorbents and evaluating the uniformity of particle distribution. Figure 2a presents the well-dispersed arrangement of ZnAl nanosheets with a layered hexagonal-shaped structure [30]. The sheets are thin, exhibiting uniform thickness and lateral dimensions at the nanometer scale [31]. This layered morphology is characteristic of ZnAl-LDH, confirming the successful synthesis of the material. Furthermore, the absence of significant agglomeration indicates homogeneous dispersion [23]. Figure 2b represents a starch–ZnAl composite and reveals a well-defined hybrid structure, where ZnAl nanosheets are integrated with starch matrices [32]. The amorphous regions surrounding the ZnAl layers indicate the presence of starch, which enhances the overall stability, structural integrity, and reduces the tendency of the biocomposite nanosheets to restack [25,33]. The nanosheets retain their characteristic thinness and layered morphology, suggesting effective interactions between starch and ZnAl [34].

Overall, TEM analysis represents the successful fabrication of a well-dispersed ZnAl–starch composite with a stable layered architecture, which enables favorable adsorption applications [23].

#### 3.1.3. XRD Analysis

**Figure 3:**
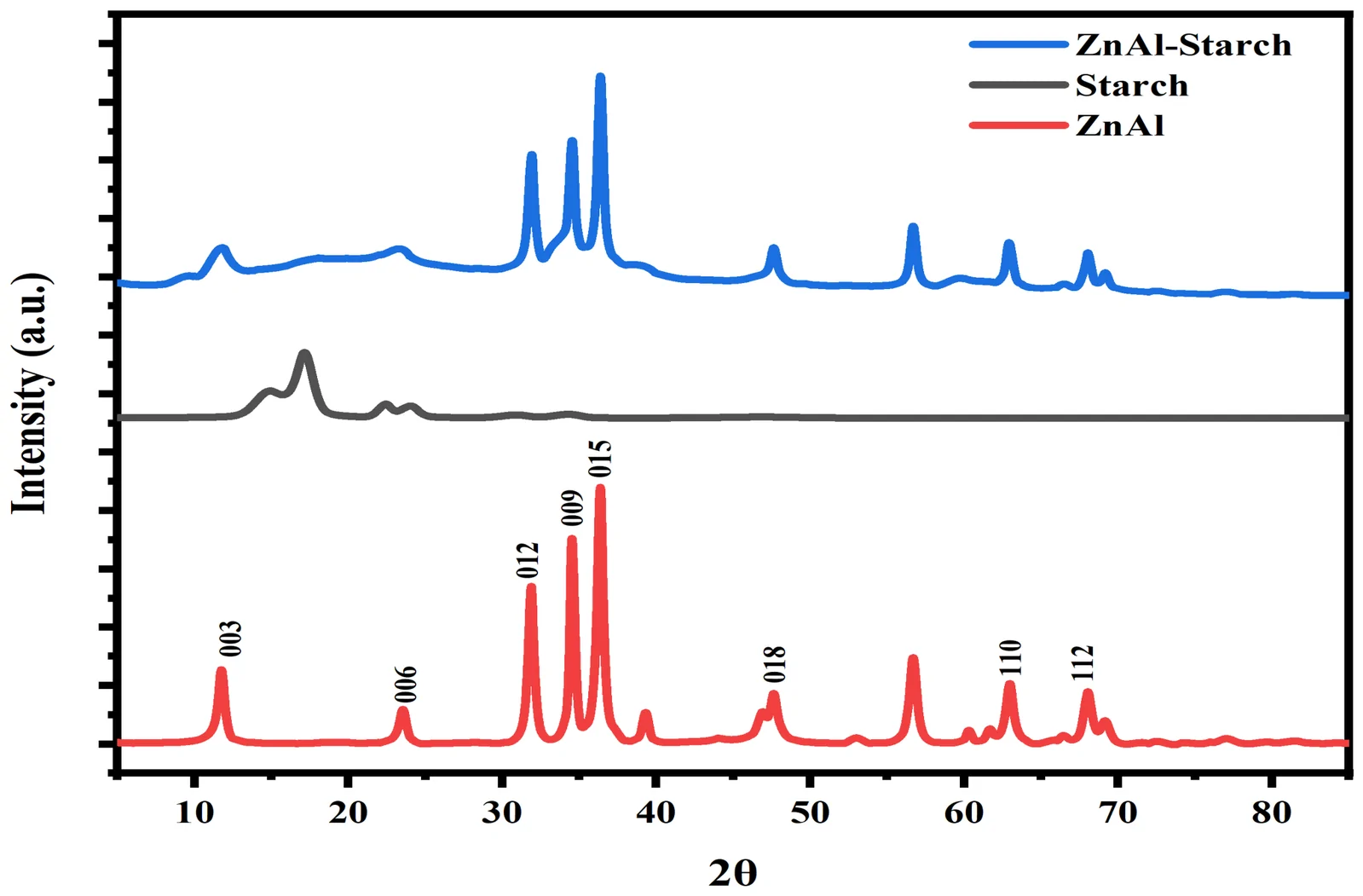
XRD image of ZnAl and starch–ZnAl composites.

The X-ray diffractions (XRDs) of the synthesized, pristine ZnAl layered double hydroxide (ZnAl), pure starch, and the composite (ZnAl–starch) are shown in Figure 3. The diffraction peaks of the ZnAl sample are very sharp and symmetrical, and appear at the 2θ positions characteristic of planes, including (003), (006), (110), and (112), which are typical of the hydrotalcite-like, crystalline, layered structure reported for ZnAl-LDH systems [23]. The sharp peaks of ZnAl widened because of the amorphous phase of the starch intercalated between the ZnAl nanosheets, as shown in the TEM in Figure 2b. In the case of pure starch, there are broad characteristic diffraction peaks at a range of 15–25°, which are characteristic of the composite’s semi-crystalline nature. The basal reflections of the LDH phase are maintained when the LDH is integrated into the ZnAl–starch composite, which shows that the integrity of the LDH layered matrix is maintained during the composite fabrication process. The minor shifts and variations in the intensity of the peaks suggest a good physical and chemical interaction between the hydroxyl groups of the starch macromolecule and the metal hydroxide layers of the LDH, which is confirmed by similar biopolymer–LDH composite systems [35].

#### 3.1.4. FTIR Analysis

**Figure 4:**
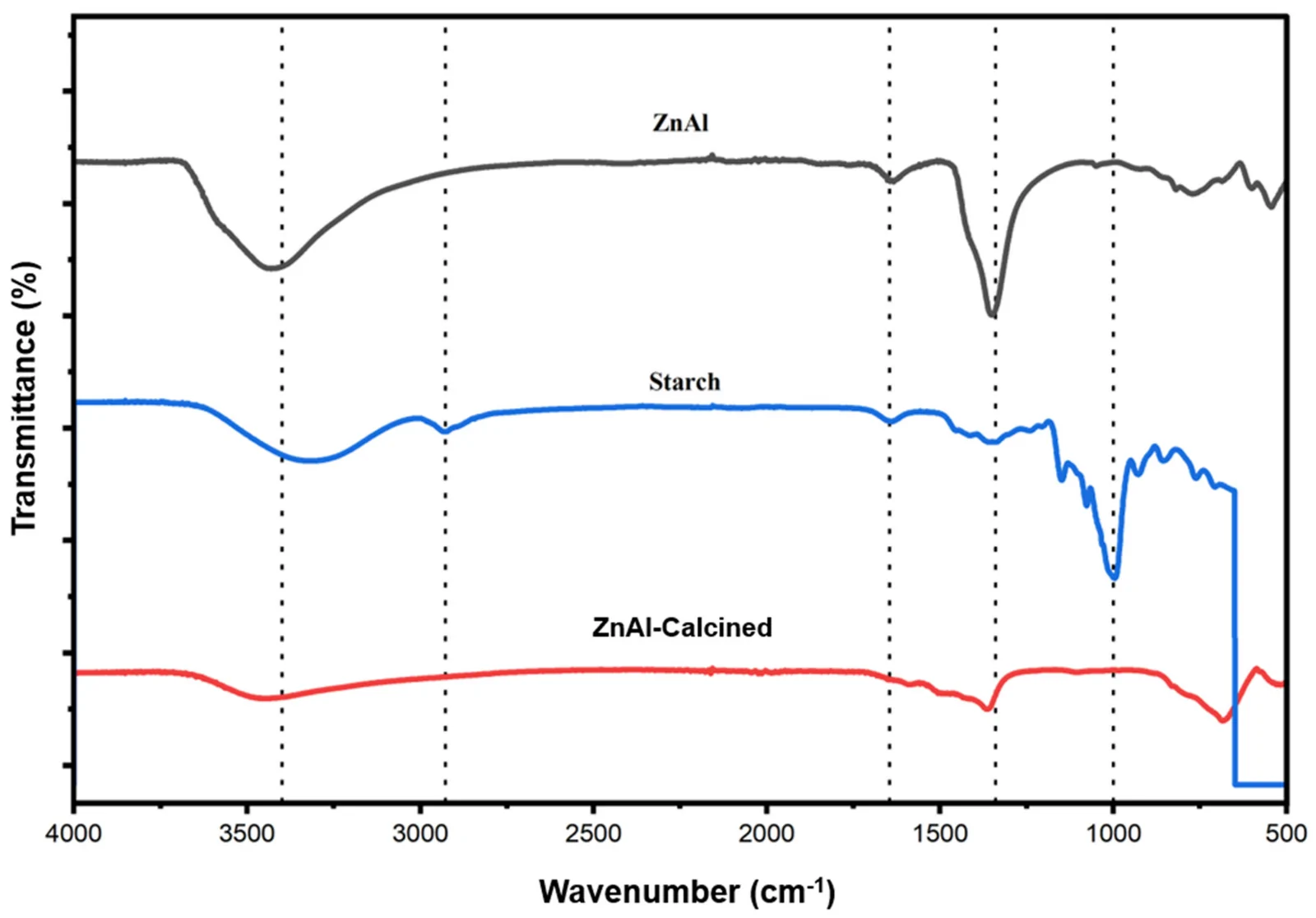
FTIR images of a ZnAl, ZnAl−starch, and ZnAl−starch calcined composites.

The functional groups of the as-developed adsorbents were analyzed using a FTIR test as presented in our previous studies. The Fourier transform infrared (FTIR) spectra of ZnAl-LDH, pure starch, and calcined composites are shown in Figure 4. The broad absorption band at about 3400 cm^−1^ is attributed to the stretching band of hydroxyl (-OH) groups of both interlayer water molecules and brucite-like metal hydroxide sheets. The band near 1630 cm^−1^ is associated with the bending vibrations of the adsorbed water molecules. The characteristic peaks identified in the spectrum of pure starch are located around 2920 cm^−1^ (C–H stretching) and in the range of 1000–1200 cm^−1^ (C–O and C–O–C stretching of the saccharide ring structure). In the ZnAl–starch composite profiles, these characteristic saccharide vibrations are overlaid by the appearance of the specific metal–oxygen (Zn–O and Al–O) lattice vibrations below 800 cm^−1^ [30]. A significant amount of shifting and broadening of the hydroxyl and ether linkage bands can be observed, which is indicative of strong hydrogen bonding interactions occurring between the starch biopolymer matrix and inorganic ZnAl-LDH interface, consistent with the structure characterization of functionalized hybrid materials [36].

### 3.2. RSM Models for Acid Blue Uptake

The experimental results based on the design variables are shown in Table 2. The adsorption experiments were designed according to a response surface methodology (RSM), and the experimental runs were randomized to minimize the influence of uncontrolled variables. Regression analysis was initially performed using a full quadratic polynomial model. To improved model accuracy and reliability while avoiding any biasness, the statistically insignificant higher-order and interaction terms (*p* > 0.05) were eliminated to obtain simplified regression models without compromising the predictive capability of the models while ensuring all main effect terms were retained. The adequacy of the reduced models was subsequently evaluated using analysis of variance (ANOVA), coefficient of determination (R^2^, adjusted R^2^, and predicted R^2^), adequate precision, and lack-of-fit tests. The final reduced empirical models, expressed in terms of coded variables (A, B, and C) for the residual Acid Blue concentration after adsorption onto ZnAl-LDH, ZnAl-C, ZnAl-LDH-S, and ZnAl-LDH-CS, are given by Equations (3)–(6), respectively.

ZnAl-LDH Residual conc. (mg/L) = +41.95 + 22.37A + 9.71B + 2.1C − 11.83B^2^ (3)

ZnAl-LDH-C, Residual conc. (mg/L) = +38.10 + 22.77A + 8.36B + 2.07C + 3.83AC + 4.42BC − 5.67B^2^ (4)

ZnAl-LDH-S, Residual conc. (mg/L) = +37.55 + 23.84A + 3.72B + 2.22C + 4.65AC + 4.69BC(5)

ZnAl-LDH-CS, Residual conc. (mg/L) = +31.92 + 21.36A + 3.85B + 0.1624C + 3.44BC − 8.40A^2^ + 5.59C^2^ (6)

**Figure 5:**
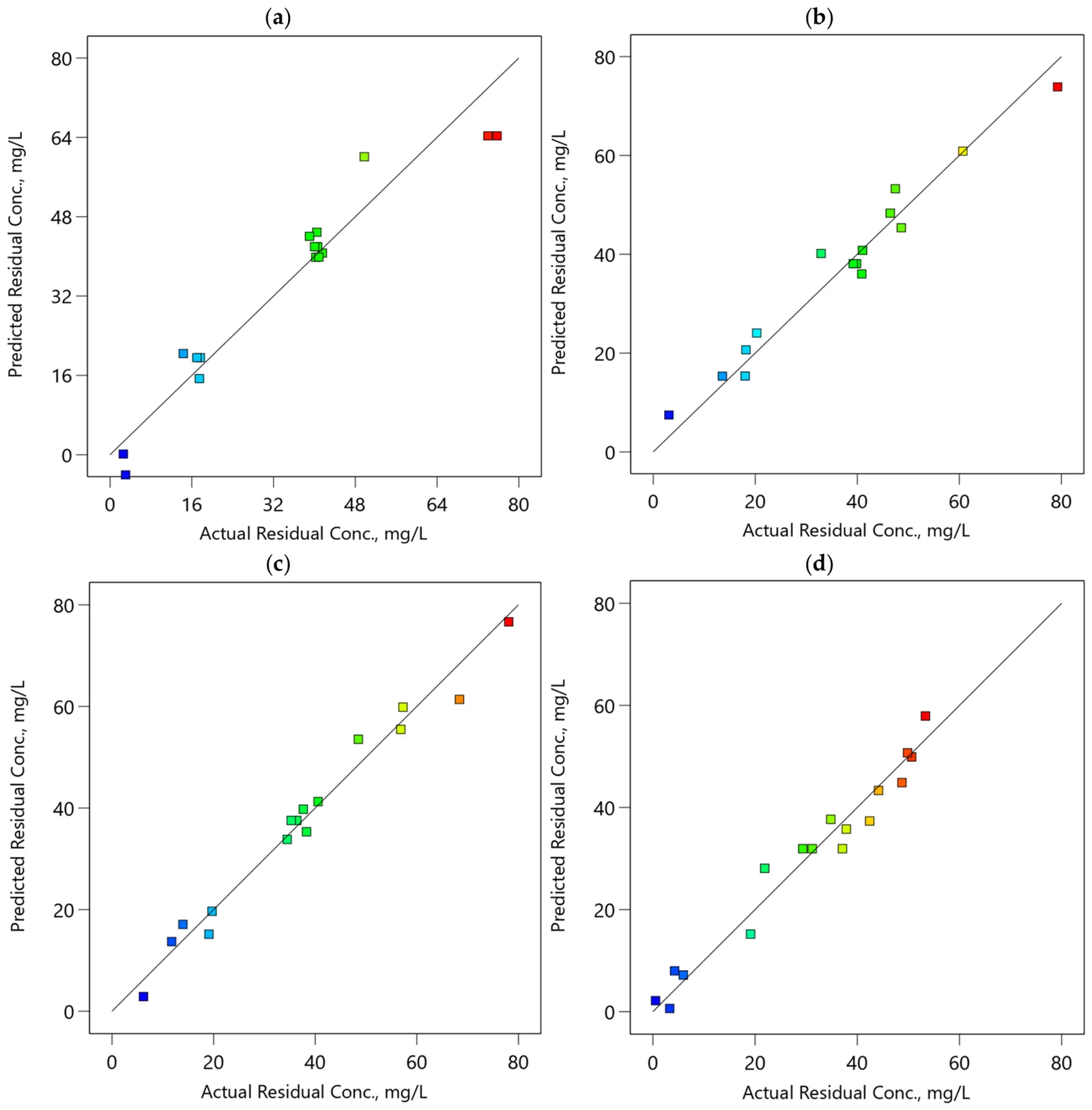
Actual versus predicted values of Acid Blue residual concentration after adsorption onto (**a**) ZnAl-LDH, (**b**) ZnAl-C, (**c**) ZnAl-LDH-S, and (**d**) ZnAl-LDH-CS.

Table 3 summarizes the ANOVA results for the reduced response surface models. The simplified regression models developed for ZnAl-LDH and ZnAl-C were both highly significant (*p* < 0.0001), with F-values of 39.92 and 41.31, respectively, confirming that the selected variables adequately describe the adsorption process. For ZnAl-LDH, the significant model terms were the linear effects of initial dye concentration (A) and pH (B), together with the quadratic effect of pH (B^2^), whereas the linear effect of adsorbent dosage (C) was statistically insignificant. Likewise, the ZnAl-C model retained the linear effects of A and B, the interaction terms AC and BC, and the quadratic term B^2^ as significant variables, while the linear effect of C remained insignificant. The statistical parameters listed in Table S1 further demonstrate the adequacy of both models. The ZnAl-LDH model exhibited R^2^, adjusted R^2^, and predicted R^2^ values of 0.9301, 0.9068, and 0.8378, respectively, whereas the corresponding values for ZnAl-C were 0.9612, 0.9379, and 0.7817. In addition, adequate precision values of 19.66 and 24.28, were substantially greater than the minimum desirable value of 4, confirming that both reduced models provided adequate signal-to-noise ratios for prediction and optimization. The close agreement between the experimental and predicted responses shown in Figure 5c,d further verifies the reliability of the simplified regression models.

Similarly, the reduced response surface models for ZnAl-LDH-S and ZnAl-LDH-CS were also highly significant (*p* < 0.0001), with F-values of 91.56 and 44.28, respectively (Table 3). For ZnAl-LDH-S, the adsorption response was primarily governed by the linear effect of initial dye concentration (A), followed by the interaction effects AC and BC, while pH (B) exerted a comparatively smaller but statistically significant influence. In contrast, the ZnAl-LDH-CS model retained the linear effects of A and B, the interaction term BC, and the quadratic terms A^2^ and C^2^ as significant variables. As summarized in Table S1, ZnAl-LDH-S exhibited the highest predictive performance among all models, with R^2^, adjusted R^2^, and predicted R^2^ values of 0.9765, 0.9659, and 0.9089, respectively, together with an adequate precision ratio of 33.69. Likewise, the ZnAl-LDH-CS model demonstrated excellent agreement between the experimental and predicted responses, yielding R^2^, adjusted R^2^, and predicted R^2^ values of 0.9637, 0.9420, and 0.8918, respectively, with an adequate precision value of 20.53. The excellent agreement between the experimental observations and model predictions presented in Figure 5c,d confirms that both simplified models accurately describe the adsorption behavior and are suitable for response prediction within the investigated design space.

The lack-of-fit test was further employed to evaluate the applicability of the reduced regression models. As shown in Table 3, the ZnAl-LDH, ZnAl-C, and ZnAl-LDH-S models exhibit significant lack-of-fit results (*p* < 0.05), suggesting that minor unexplained variations remain after removing statistically insignificant higher-order terms. Nevertheless, these models maintained high coefficients of determination, close agreement between the adjusted and predicted R^2^ values, and adequate precision ratios substantially exceeding the recommended threshold of 4 (Table 3), indicating satisfactory predictive capability within the experimental region. In contrast, the ZnAl-LDH-CS model exhibited a non-significant lack-of-fit result (F = 1.19, *p* = 0.5333), demonstrating excellent agreement between the experimental observations and the predicted responses. Overall, the statistical analyses confirm that the simplified regression equations retain high predictive accuracy while providing more parsimonious and interpretable models than the original full quadratic equations.

**Figure 6:**
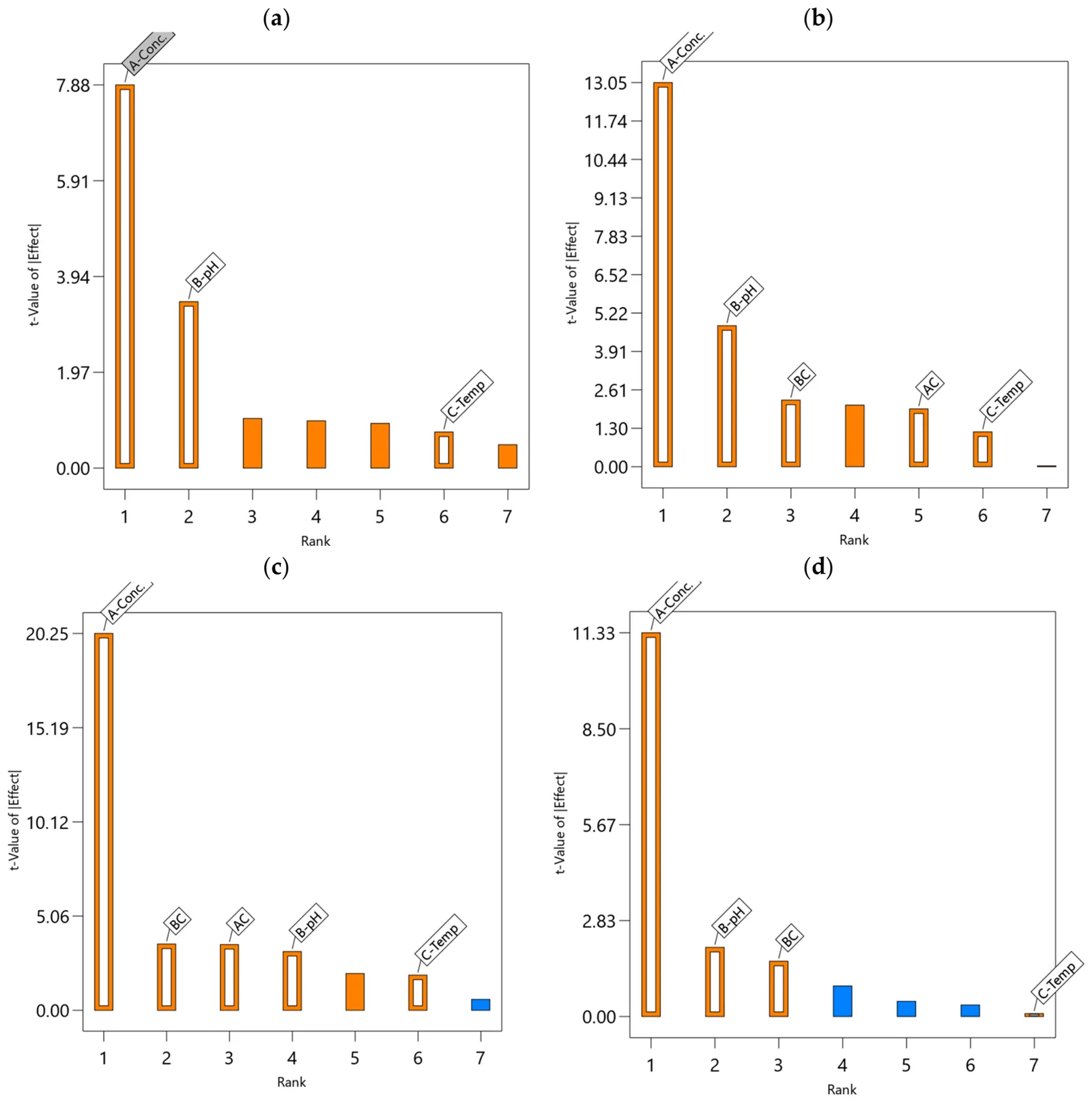
Pareto plots of the reduced models for adsorption of Acid Blue onto (**a**) ZnAl-LDH, (**b**) ZnAl-C, (**c**) ZnAl-LDH-S, and (**d**) ZnAl-LDH-CS.

The Pareto plots (Figure 6) support the ANOVA results and the reduced regression models by confirming that the initial dye concentration (A) was the dominant factor affecting the residual Acid Blue concentration for all four adsorbents. However, the relative importance of the remaining terms varied considerably among the materials. For ZnAl-LDH (Figure 6a), solution pH (B) was the second most influential factor, while the quadratic temperature term (C^2^) showed a smaller but significant contribution, consistent with the reduced model containing A, B, C, and B^2^. For ZnAl-LDH-C (Figure 6b), the response was influenced by a broader combination of factors, with B, BC, AC, and C^2^ contributing after A, in agreement with the more complex model containing both linear and interaction terms. For ZnAl-LDH-S (Figure 6c), BC and AC interactions, followed by B and C^2^, were the most influential terms after A, indicating that the adsorption response depended appreciably on interactions between the operating variables. In contrast, ZnAl-LDH-CS (Figure 6d) showed a comparatively simpler response, dominated by A, followed by smaller contributions from B and BC, while the remaining terms had relatively minor effects.The Pareto analysis demonstrates that although initial dye concentration consistently governs the adsorption response, modification of ZnAl-LDH altered the relative contributions and interactions of pH and temperature. This behavior is consistent with the reduced regression models (Equations (3)–(6)), which retain only the statistically relevant linear, interaction, and quadratic terms for each adsorbent. The removal of statistically insignificant higher-order terms therefore simplifies the models without substantially compromising their predictive capability, while also providing a clearer interpretation of the key factors controlling Acid Blue adsorption.

### 3.3. Influence of Independent Variables on the Adsorption of Acid Blue 92

**Figure 7:**
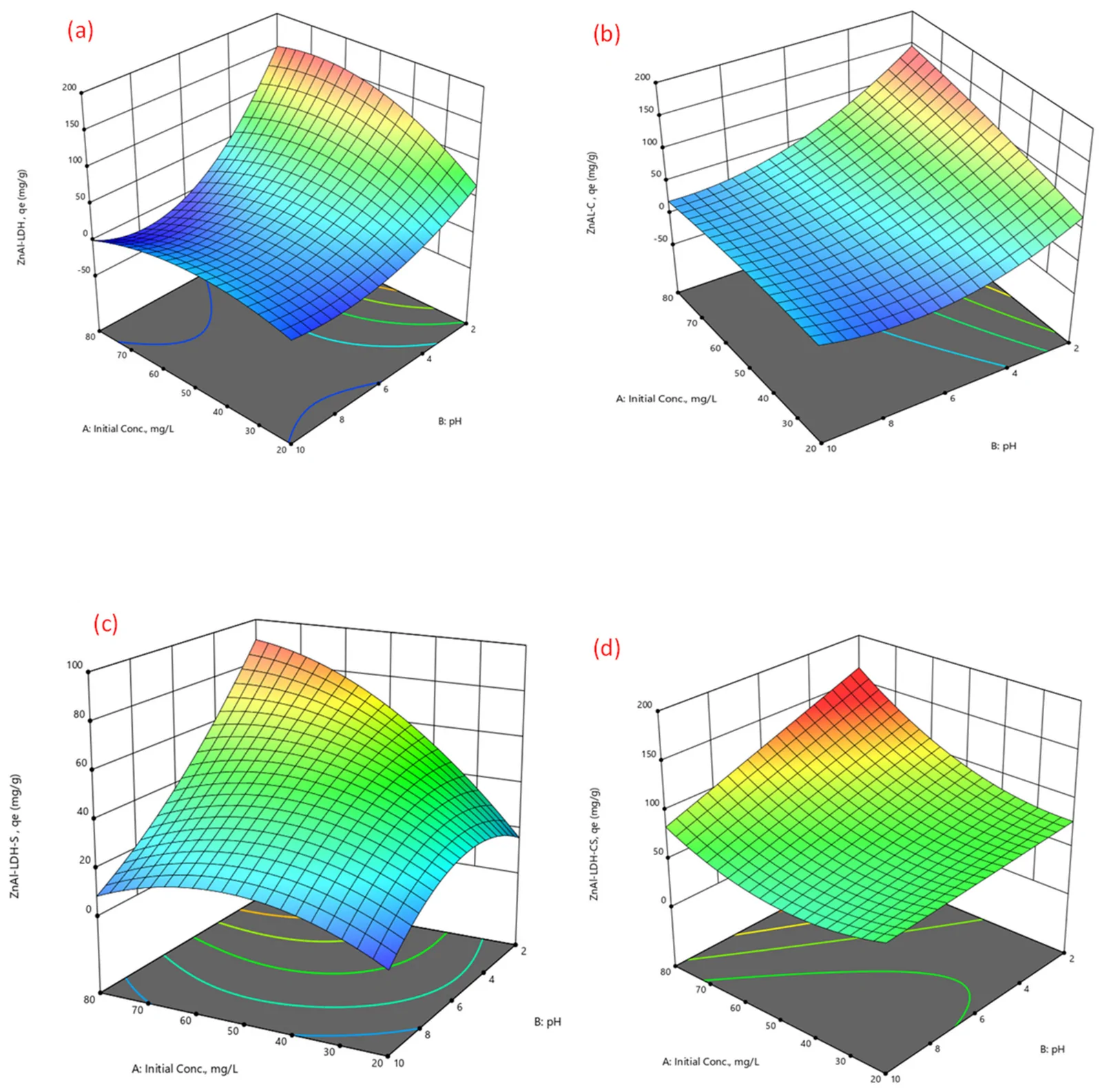
Effect of initial concentration and pH on Acid Blue adsorption by (**a**) ZnAl−LDH, (**b**) ZnAl−C, (**c**) ZnAl−LDH-S, and (**d**) ZnAl−LDH−CS.

Three-dimensional plots were generated to elucidate the influence of process variables—considering two factors per graph—on the predicted dye uptake and to identify optimal or near-optimal regions for adsorption. The combined effect of the initial dye concentration (C_0_ = 20–80 mg/L) and pH (2–10) on Acid Blue uptake is demonstrated in Figure 7. The interaction between C_0_ and pH for dye uptake is statistically significant (*p* < 0.05) for all the adsorbents (Table 3). The adsorption capacities generally reduced with the increase in pH for all the adsorbents, especially at a high initial concentration (>60 mg/L). The same trend was observed at lower concentrations for ZnAl-LDH, ZnAl-C, and ZnAl-LDH-CS. However, for ZnAl-LDH-S, q_e_ increased when the pH increased from 2 to 6, and thereafter, it dropped. The surfaces of adsorbents are highly protonated in acidic medium fostering an electrostatic attraction with the anionic dye. As the pH increases, the surfaces of the adsorbents become more negatively charged, reducing the attraction and subsequent adsorption of the dye molecules. It can be observed that uptake, across all the four adsorbents, increased as the initial dye concentration increased, especially in the low-pH region (pH < 4). This is in good agreement with the assumption that increased dye-molecule densities in the solution result in greater adsorption capacities, as more dye molecules are captured by the active sites of the LDH [37,38]. At a higher pH (pH > 4), q_e_ increased with an increase in C_0_ for ZnAl-C and ZnAl-LDH-CS, whereas q_e_ increased until C_0_ reached about 60 mg/L before decreasing for ZnAl-LDH and ZnAl-LDH-S.

**Figure 8:**
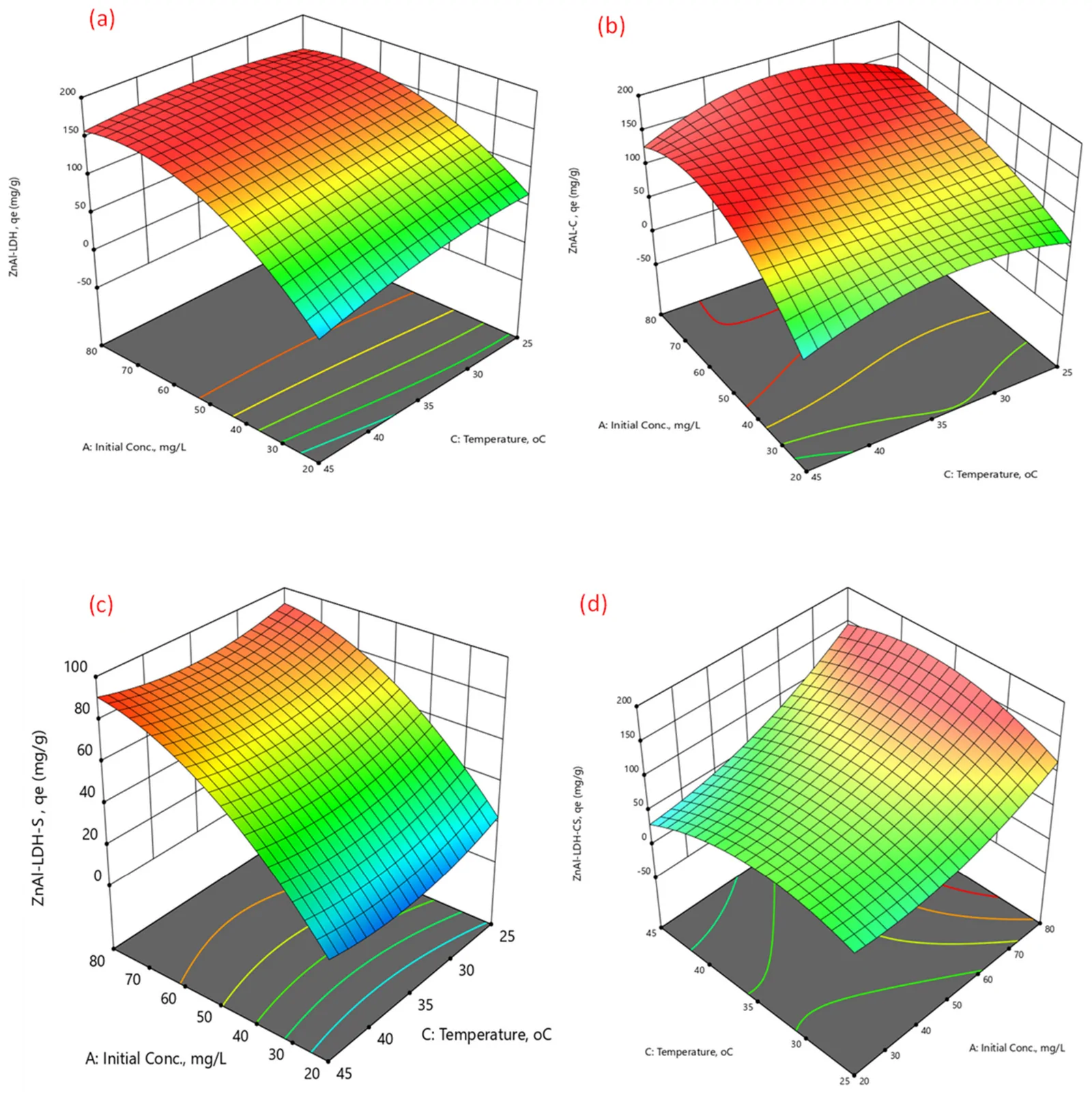
Effect of initial concentration and temperature on Acid Blue adsorption by (**a**) ZnAl-LDH, (**b**) ZnAl-C, (**c**) ZnAl-LDH-S, and (**d**) ZnAl-LDH-CS.

The correlation between the initial concentration (20–80 mg/L) and temperature (25–45 °C) is presented in Figure 8, and the interaction was insignificant for ZnAl-C (Table 3) and ZnAl-LDH-S. Across ZnAl-LDH, ZnAl-C, and ZnAl-LDH-CS, q_e_ initially increased when the temperature increased from 25 to 35 °C and later decreased when the temperature increased beyond 35 °C. In the initial phase (25 to 35 °C), the adsorbate experienced faster diffusion and increased molecular mobility, which helped the molecules gain energy to chemically bond with the surface of the adsorbate. Beyond this (temperature > 35 °C), desorption prevails as adsorption becomes exothermic. In this case, the free energy change (ΔG = ΔH − TΔS) becomes less negative as the temperature increases. For ZnAl-LDH-S, it can be observed that q_e_ reduced when the temperature increased (from 25 to 35 °C) before decreasing. For this case, physisorption initially prevails, where thermal energy overcomes weak van der Waals forces, and later, chemisorption prevails as more dye molecules gain energy to form chemical bonds with the surface of the adsorbent.

**Figure 9:**
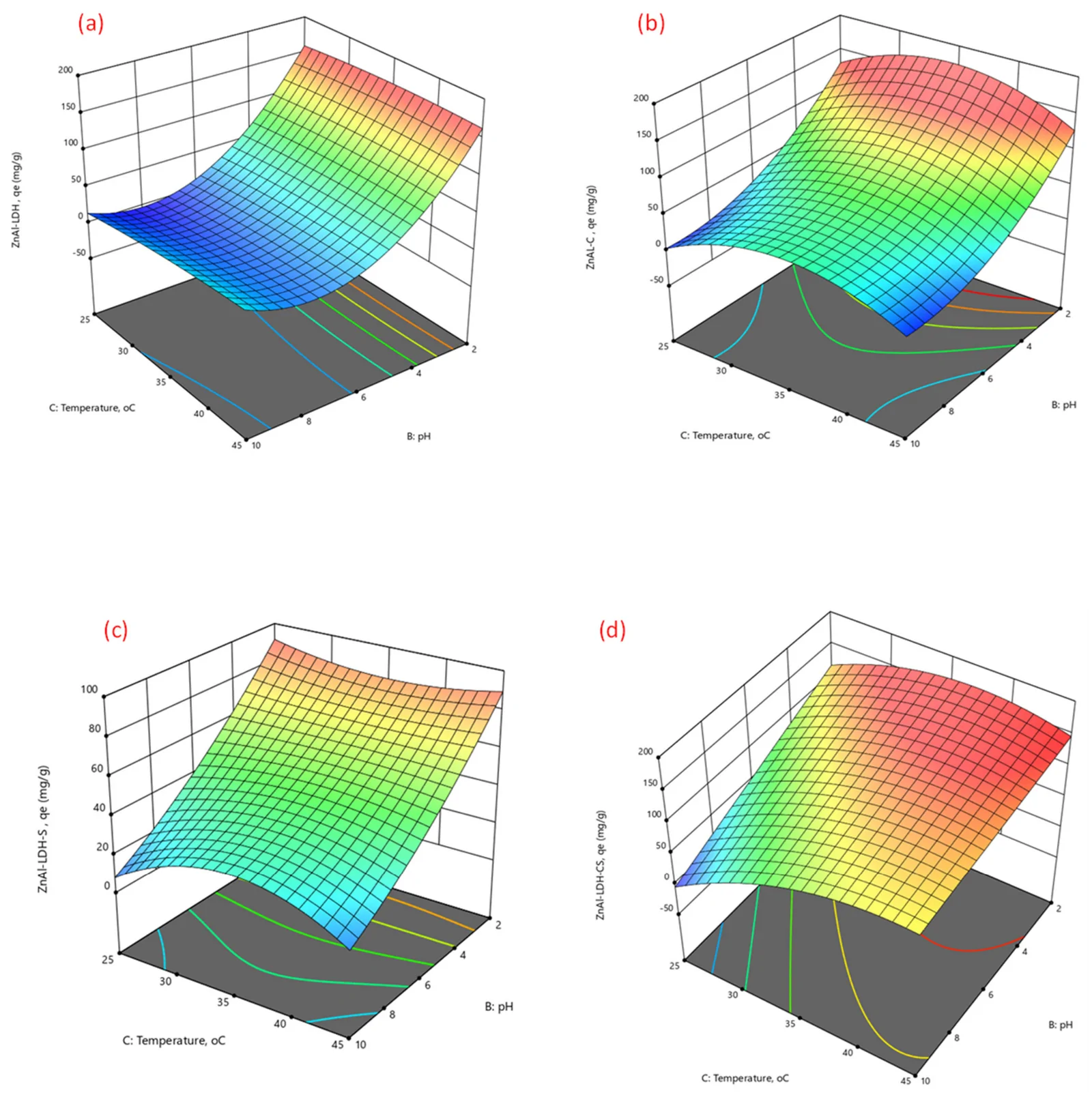
Effect of pH and temperature on Acid Blue adsorption by (**a**) ZnAl-LDH, (**b**) ZnAl-C, (**c**) ZnAl-LDH-S, and (**d**) ZnAl-LDH-CS.

Figure 9 shows the surface plot of interaction between pH and temperature for the adsorption of Acid Blue. This combined effect (BC) was not significant on the uptake of Acid Blue for the adsorbents, except for ZnAl-LDH (*p* = 0.0032). Irrespective of the temperature, the adsorption capacity decreased when the pH increased across all the adsorbents.

### 3.4. Kinetic Modeling

The adsorption mechanism of Acid Blue 92 dye molecules in ZnAl and starch-ZnAl composites was evaluated by fitting adsorption kinetic data onto widely adopted kinetic models: pseudo-first-order and pseudo-second-order models. The kinetic parameters and goodness-of-fit statistics are presented in Table 4. The nonlinear PFO model described all four kinetic profiles more accurately than PSO, yielding higher $R^{2}$ values (0.9947–0.9968), lower RMSE values (2.25–4.10 mg g^−1^), and markedly smaller $q_{e}$ deviations (4.82–13.65%). In contrast, PSO overestimated equilibrium uptake by a range of 36.94–63.15%, producing systematically larger SSE and RMSE values despite $R^{2}>0.989$. ZnAl-LDH exhibited the highest experimental equilibrium capacity (159.20 mg g^−1^) and the largest PFO rate constant ($k_{1}=0.01074$ min^−1^), followed by ZnAl-LDH-C, ZnAl-LDH-CS, and ZnAl-LDH-S. The $k_{1}$ values of 0.009194, 0.007489, and 0.006805 min^−1^ indicate progressively slower apparent approaches to equilibrium. Starch incorporation therefore reduced the adsorption rate and equilibrium uptake under the specific conditions investigated, while the calcination of the starch-containing composite partially restored both responses. These trends may reflect changes in site accessibility, pore connectivity, and dye transport arising from starch incorporation and thermal treatment [36,39,40].

**Figure 10:**
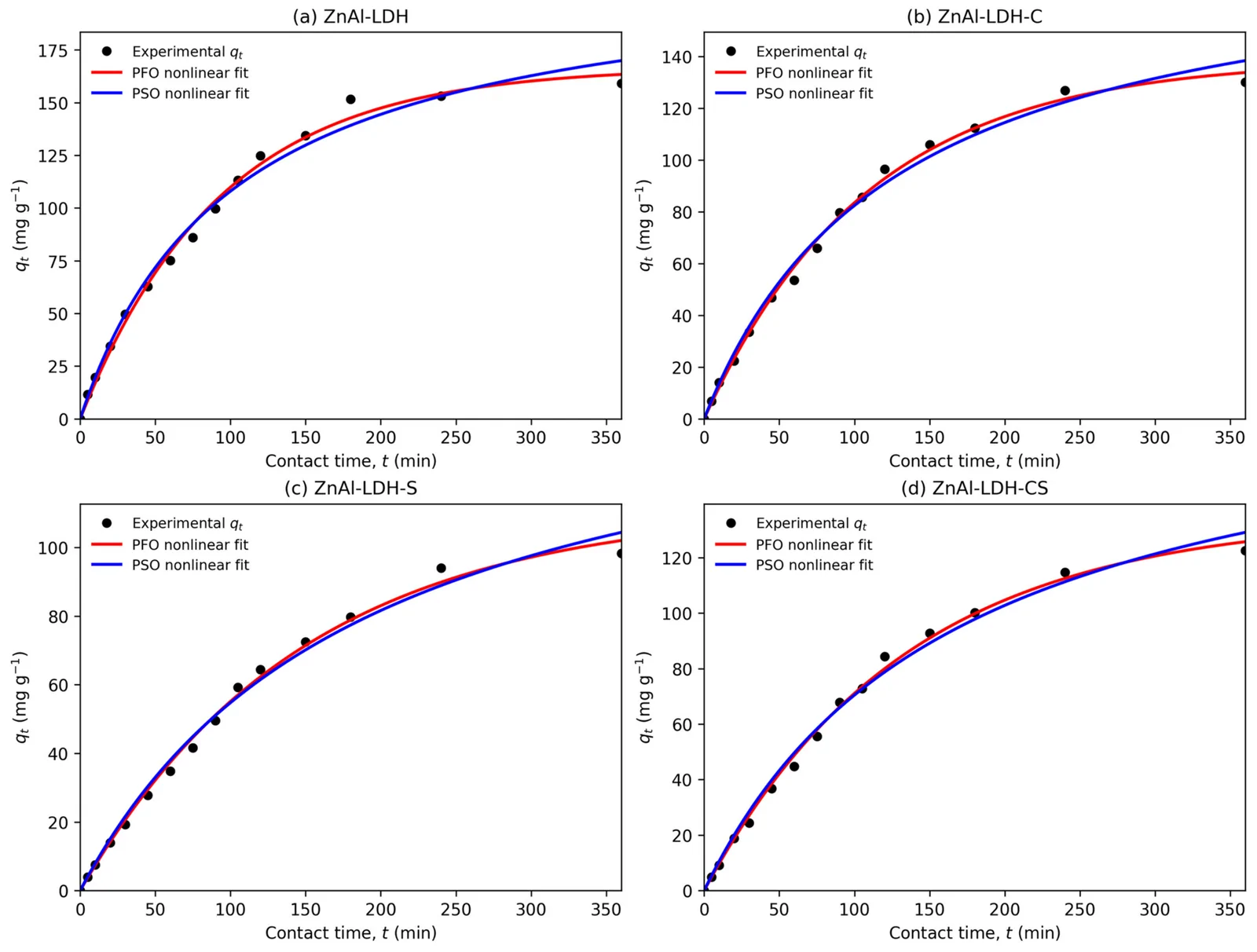
Experimental adsorption kinetics and nonlinear pseudo-first-order (PFO) and pseudo-second-order (PSO) model fits for Acid Blue 92 adsorption onto ZnAl and ZnAl-starch composites.

Figure 10 compares the experimental $q_{t}$ values with the nonlinear PFO and PSO predictions for each adsorbent. As illustrated in Figure 10, all four adsorbents exhibited a rapid initial increase in Acid Blue 92 uptake, followed by a progressively slower approach towards equilibrium. The initially rapid uptake may be attributed to the relatively high concentration gradient between the bulk solution and the adsorbent surface and to the availability of readily accessible adsorption sites [41,42]. As these sites became increasingly occupied, the adsorption rate decreased, producing the characteristic asymptotic kinetic profiles observed for all materials.

Accordingly, the kinetic results support an apparent first-order uptake regime governed by material-dependent site accessibility and mass-transfer behavior. The nonlinear PFO model provides the closest description of Acid Blue 92 uptake but does not establish the nature of the adsorbate–surface interaction. The pronounced pH dependence and alkaline regenerability are consistent with a substantial reversible contribution involving electrostatic association and other non-covalent interactions at accessible LDH and starch-containing domains. However, because the available FTIR spectra characterize the materials before adsorption and no post-adsorption XPS, FTIR, or activation-energy measurements were obtained, the present data do not permit an unambiguous distinction between physisorption and chemisorption. The kinetic models are therefore interpreted as empirical descriptions of uptake behavior rather than molecular evidence of a specific adsorption mechanism [43,44]. Conclusively, although nonlinear kinetic analysis showed that the PFO model described the kinetic data better, the overall kinetic modeling findings suggest that Acid Blue 92 adsorption is governed by a combination of physicochemical interactions, indicating a complex adsorption mechanism is involved.

### 3.5. Regeneration Performance

**Figure 11:**
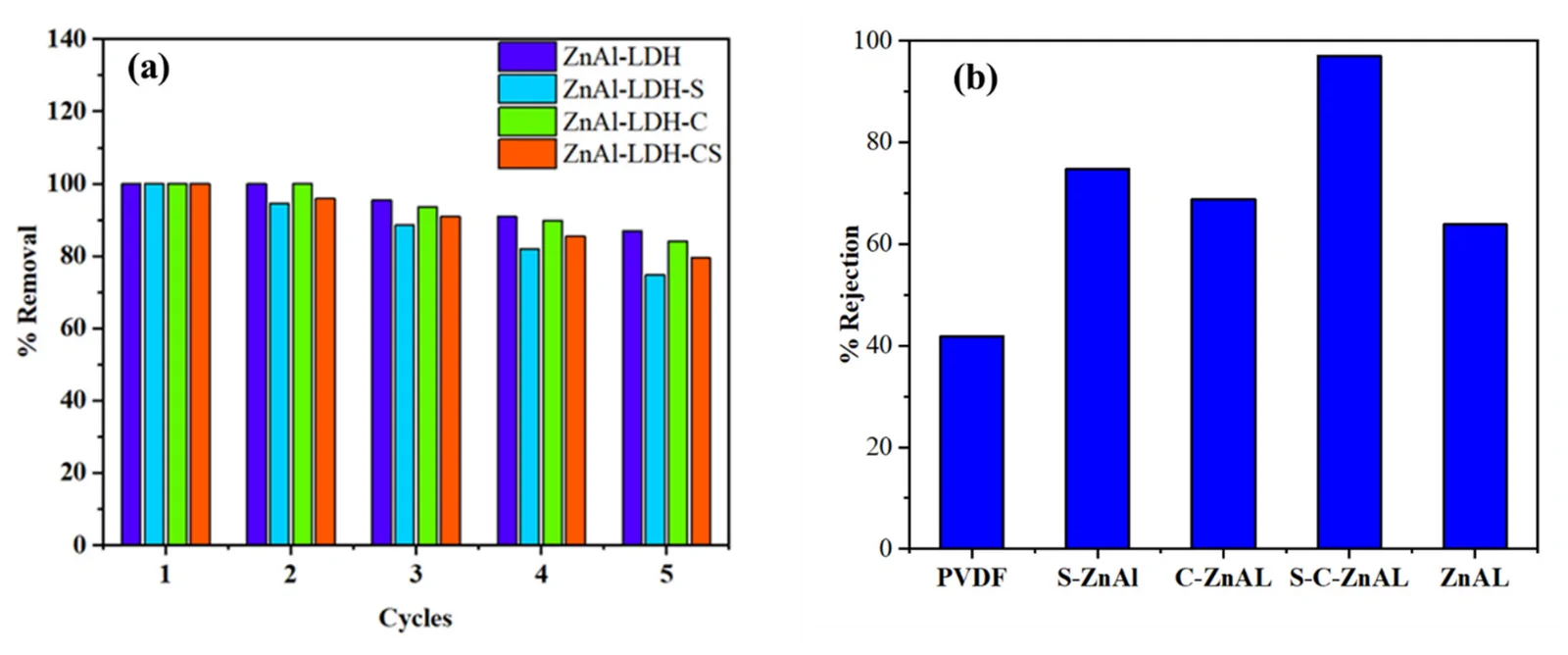
(**a**) Regeneration performance of ZnAl and starch–ZnAl composites; (**b**) % rejection of Acid Blue 92 dye in PVDF-based ZnAl and starch ZnAl composite membranes.

The recyclability of the adsorbent is a key indicator for potential industrial application. To evaluate regeneration performance, the prepared adsorbents are investigated for five-consecutive recyclability cycles, and the results are depicted in Figure 11a. As can be seen, ZnAl and ZnAl-C show a high regeneration performance for five cycles with a reduction in adsorption efficiency in the range of 10–12%. This indicates better structural stability after regeneration mainly associated with high LDH crystallinity. Consequently, the starch–ZnAl composite showed almost 20% reduction in adsorption efficiency after regeneration for five cycles. The reduction in adsorption efficiency could be associated with starch, which resulted in the breakdown of the structure and dissolution due to low chemical stability [45]. As expected, the calcined starch–ZnAl nanocomposite showed excellent recyclability, which corresponds to a physical interaction between the dyes and adsorbent providing ease of desorption and structure stability during chemical regeneration.

### 3.6. Membrane Performance

The prepared ZnAl and starch–ZnAl composites were utilized as fillers in PVDF membranes for the enhanced filtration of Acid Blue 92 dyes molecules in wastewater. The results of membrane filtration are shown in Figure 11b. The pristine PVDF membrane shows a rejection value of almost 41%. The PVDF membrane intercalated with ZnAl, and calcined ZnAl showed improved rejection values of 60% and 70%. Interestingly, the addition of starch–ZnAl and calcined starch–ZnAl LDH to the PVDF membrane significantly increased the % rejection of Acid Blue 92 dye to 78% and 98%, respectively. The high rejection of the starch-based LDH composite membrane is attributed to the porous nanostructure and high hydrophilicity, which improved the rejection of dye molecules.

### 3.7. Membrane Hydrophilicity, Transport Performance, and Separation Mechanism

**Figure 12:**
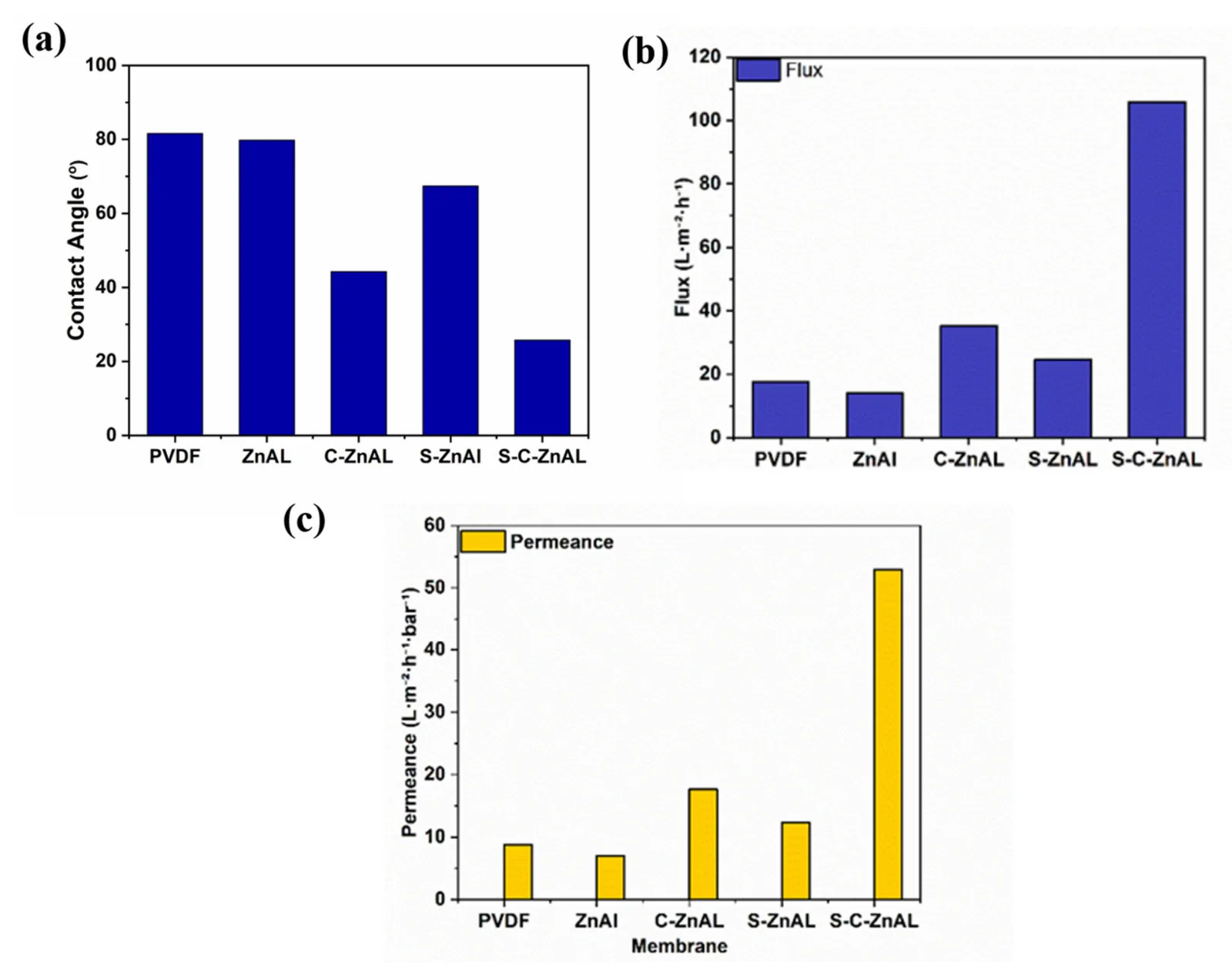
(**a**) Contact angle, (**b**) water flux, and (**c**) permeance of PVDF and PVDF–starch–LDH membranes.

The contact angle, pure water flux, and permeance of the prepared membranes were assessed under the same conditions (10 min, 2 bar, membrane area = 0.00173 m^2^) and are depicted in Figure 12. The findings demonstrate how the presence of starch–LDH and calcination majorly impacted the PVDF composite membrane’s hydrophilicity and permeability. The measured contact angles for the membranes given in Figure 12a and corresponding pictures in Table S1 show significant alterations in the affinity to water across the different membranes due to calcination, as well as the incorporation of starch in the pristine ZnAl-LDH. As shown, the raw PVDF membrane’s intrinsic hydrophobicity (81°) and constrained capacity for water transport were demonstrated by its comparatively lower flux of 17.3 L·m^−2^·h^−1^ and lower permeance of 8.7 L·m^−2^·h^−1^·bar^−1^. The addition of ZnAl LDH to the PVDF membrane resulted in improved hydrophilicity with a water contact angle of 79.8°, but showed the lowest water transport result compared to all the membranes that were evaluated (permeance: 6.9 L·m^−2^·h^−1^·bar^−1^, flux: 13.8 L·m^−2^·h^−1^). This indicates inadequate interfacial contact and LDH particle aggregation within the membrane matrix, which leads to pore-blocking and reduced water permeation [46]. Abdollahi et al. reported that a high concentration of MgAl LDH resulted in a decrease in the number of pores and their size structure [47]. In contrast, the addition of a starch-intercalated ZnAl (S-ZnAl) membrane reduced the contact angle to 67.4°, and thereby significantly improved the water transport performance with a flux of 24.2 L·m^−2^·h^−1^ and a permeance of 12.1 L·m^−2^·h^−1^·bar^−1^. The improved water-membrane affinity and greater hydrophilicity were attributed to the presence of starch hydroxyl groups [48]. Interestingly, the calcined ZnAl (C-ZnAl) membrane revealed a more noticeable effect, where the contact angle decreased to 44.3°, the flux increased to 69.0 L·m^−2^·h^−1^, and permeance reached 34.5 L·m^−2^·h^−1^·bar^−1^. The calcination of ZnAl LDH resulted in a porous and disrupted ZnAl structure. Therefore, coupling calcined ZnAl in the PVDF structure improved the porosity and formed nanochannels, which facilitated easy water passage [38]. In addition, good hydrophilicity and water transport performance were obtained with the synergistic modification in S-C-ZnAl, with a water contact angle of 25.8°, flux of 103.6 L·m^−2^·h^−1^, and permeance of 51.8 L·m^−2^·h^−1^·bar^−1^. This implies that when starch modification was combined with calcination (S-C-ZnAl), the performance significantly peaked with the flux and permeance, improving by 42.9% and 25%, respectively, over C-ZnAl, while dye rejection also markedly increased by 35.7% [49]. This improved behavior suggests that the calcination of starch–ZnAl resulted in a highly porous carbon-mixed metal-oxide framework, as confirmed from the SEM analysis, which then intercalated as a nanofiller in the PVDF matrix, which greatly improved the water affinity and transport channels, outperforming the virgin and single-modified membranes [50]. The outcomes clearly show a hierarchy of the permeability performance, S-C-ZnAl > C-ZnAl > S-ZnAl > PVDF > ZnAl, while the order for dye rejection was S-C-ZnAl > S-ZnAl > C-ZnAl > ZnAl > PVDF. The results demonstrate the effectiveness of the starch modification approach, especially dual-component doping, in enhancing membrane permeability, which makes S-C-ZnAl a very viable option for high-performance filtration applications [35]. Accordingly, the results affirm that starch modification enhances dye affinity and dispersion, while calcination improves structural integrity and pore accessibility. The synergistic effect of both treatments for the S-C-ZnAl composites leads to superior membrane performance, making them promising candidates for high-efficiency wastewater treatment.

Within the limits of the available measurements, Acid Blue 92 rejection is best interpreted as a coupled filler-mediated adsorption and size-exclusion process rather than a single-pathway separation mechanism [51,52]. The approximately 41% rejection obtained with pristine PVDF represents the baseline retention by the unmodified membrane, whereas the progressive increase to a range of approximately 60–98% following the incorporation of ZnAl-based nanofillers demonstrates an additional filler-dependent contribution (Figure 11b). PVDF–LDH and other adsorptive PVDF mixed-matrix membranes have been shown to capture dyes at accessible nanofiller domains while sustaining appreciable water permeation, supporting adsorption as the most plausible dominant contributor to the improvement relative to pristine PVDF [51,53]. Notably, the S-C-ZnAl membrane combined the highest Acid Blue 92 rejection rate with the highest permeance and the lowest contact angle (Figure 12), indicating that the enhanced rejection cannot be explained solely by the formation of smaller or more restrictive pores; nevertheless, size exclusion is expected to contribute concurrently through the porous membrane matrix [52,53]. Because of the inability to measure the membranes’ zeta potential and pore-size or molecular-weight-cut-off distributions during this study, the relative contributions of electrostatic and steric exclusion cannot be quantitatively resolved, and charge repulsion cannot be assigned as the dominant rejection mechanism [52,54].

## 4. Conclusions

Novel starch-modified ZnAl layered double-hydroxide (Starch–ZnAl-LDH) nanocomposites in both calcined and uncalcined forms, together with their corresponding PVDF mixed-matrix membranes, were successfully synthesized via co-precipitation and phase inversion techniques. This study demonstrates, for the first time, the development of a multifunctional material platform capable of serving both as a recyclable adsorbent and as an efficient membrane nanofiller for the recovery of dye-contaminated water. Structural characterization by SEM, TEM, XRD, and FTIR confirmed the successful incorporation of starch into the ZnAl-LDH framework, producing well-dispersed layered nanostructures with crystalline ZnAl phases embedded within a partially amorphous starch matrix and abundant surface functional groups favorable for pollutant adsorption.

Adsorption performance was strongly governed by solution pH, temperature, and initial dye concentration. Acid Blue 92 uptake was described by the nonlinear PFO model, while the available pH-dependent and regeneration results support substantial reversible dye–surface interactions. Among the investigated materials, the calcined starch-modified composite exhibited the best overall performance by combining high adsorption capacity, excellent regeneration stability, and enhanced structural accessibility. The composites maintained good recyclability, with only a minor reduction in adsorption efficiency after repeated adsorption–desorption cycles, highlighting their potential for long-term practical application. Beyond adsorption, the incorporation of starch–ZnAl-LDH nanocomposites into PVDF membranes substantially enhanced membrane performance, achieving Acid Blue rejection efficiencies approaching 98%, while increasing membrane flux and permeance by 42.9% and 25%, respectively. Membrane rejection was attributed to coupled filler-mediated adsorption and size exclusion, with adsorption providing the most plausible explanation for the improvement over pristine PVDF.

This work demonstrates that combining renewable starch with ZnAl layered double hydroxides and thermal activation provides an effective route to developing sustainable, recyclable, and multifunctional materials for water treatment. The resulting starch–ZnAl-LDH nanocomposite not only improves adsorption performance, but also enhances PVDF membrane separation efficiency while maintaining excellent long-term reusability. By integrating adsorption and membrane filtration into a single platform, the developed material offers a practical and resource-efficient solution for recovering dye-contaminated water. These findings highlight the potential of bio-based polymer nanocomposites for scalable water treatment applications and contribute to the development of next-generation sustainable materials that support circular water management and environmental protection.

## Figures and Tables

**Table 1:** Experimental ranges and levels of independent variables.

Independent Variables	Code		Range and Level	
		−1	0	+1
Initial dye concentration (mg/L)	A	20	50	80
pH	B	2	6	10
Temperature (°C)	C	25	35	45

**Table 2:** Design matrix of the CCD and the response for the adsorption of Acid Blue 92.

Run		Independent Variables		Removal Efficiency (%)				Adsorption Capacity (mg/g)			
	A	B	C	ZnAl-LDH	ZnAl-LDH-C	ZnAl-LDH-S	ZnAl-LDH-SC	ZnAl-LDH	ZnAl-LDH-C	ZnAl-LDH-S	ZnAl- LDH-SC
1	20	2	25	84.76	84.62	30.21	70.21	67.80	67.69	24.17	56.17
2	80	2	25	48.06	39.21	28.94	36.68	153.78	125.49	92.62	117.39
3	20	10	25	12.59	9.93	4.48	78.74	10.07	7.94	3.58	62.99
4	80	10	25	0.48	5.10	3.01	0.30	0.95	10.20	6.02	0.60
5	20	2	45	73.85	80.98	37.90	66.99	29.54	32.39	15.16	26.80
6	80	2	45	49.41	41.93	28.39	44.79	158.12	134.17	90.86	143.33
7	20	10	45	11.61	9.09	1.54	4.34	9.29	7.27	1.23	3.47
8	80	10	45	7.56	0.92	2.38	33.31	24.18	2.93	7.63	106.59
9	20	6	35	14.97	32.17	41.26	97.34	11.97	25.73	33.01	77.87
10	80	6	35	5.32	24.14	14.49	39.07	17.02	77.24	46.37	125.02
11	50	2	35	71.33	59.42	31.03	56.21	142.66	118.83	62.06	112.42
12	50	10	35	19.45	17.93	18.89	24.28	38.90	35.86	37.77	48.57
13	50	6	25	18.27	18.21	23.38	15.12	36.54	36.42	46.77	30.24
14	50	6	45	21.92	34.18	24.68	30.35	43.84	68.35	49.35	60.71
15	50	6	35	19.34	20.91	28.39	41.32	38.67	41.82	56.77	82.63
16	50	6	35	18.77	20.29	27.21	37.61	37.55	40.58	54.41	75.21
17	50	6	35	19.90	21.59	29.45	25.80	39.80	43.17	58.91	51.60

**Table 3:** ANOVA results for the response surface model for the adsorption of Acid Blue by ZnAl and starch–ZnAl composites.

	ZnAl		Calcined ZnAl		ZnAl–Starch		Calcined ZnAl–Starch	
Source	F-Value	*p*-Value	F-Value	*p*-Value	F-Value	*p*-Value	F-Value	*p*-Value
Model	39.92	<0.0001	41.31	<0.0001	91.56	<0.0001	44.28	<0.0001
A	121.65	<0.0001	202.97	<0.0001	418.34	<0.0001	241.18	<0.0001
B	22.94	0.0004	27.34	0.0004	10.16	0.0086	7.82	0.0189
C	1.08	0.3199	1.67	0.2254	3.64	0.0830	0.0139	0.9083
AB	-	-	-	-	-	-	-	-
AC	-	-	4.60	0.0577	12.73	0.0044	-	-
BC	-	-	6.11	0.0331	12.93	0.0042	5.01	0.0492
A^2^	-	-	-	-	-	-	11.30	0.0072
B^2^	14.02	0.0028	5.17	0.0463	-	-	-	-
C^2^	-	-	-	-	-	-	5.00	0.0493
Lack of fit	624.47	0.0016	305.34	0.0033	52.29	0.0189	1.19	0.5333
R^2^	0.9301		0.9612		0.9765		0.9637	
Adjusted R^2^	0.9068		0.9379		0.9659		0.9420	
Predicted R^2^	0.8378		0.7817		0.9089		0.8918	
Std. Dev.	6.41		5.06		3.69		4.35	
Adeq Precision	19.6628		24.2806		33.6946		20.5295	

**Table 4:** Nonlinear pseudo-first-order and pseudo-second-order kinetic parameters for Acid Blue 92 adsorption onto ZnAl-LDH-based composites.

Adsorbent	q_e_, Exp (mg g^−1^)	Pseudo-First-Order (PFO) Kinetic Parameter						Pseudo-Second-Order (PSO) Kinetic Parameter						
		q_e_, Cal (mg g^−1^)	k_1_ (min^−1^)	SSE (mg^2^ g^−2^)	R^2^	RMSE (mg g^−1^)	Δq_e_ (%)	q_e_, cal (mg g^−1^)	k_2_ (×10^−5^ g mg^−1^ min^−1^)	*h* (mg g^−1^ min^−1^)	SSE (mg^2^ g^−2^)	R^2^	RMSE (mg g^−1^)	Δq_e_ (%)
**ZnAl-LDH**	159.20	166.87	0.01074	218.28	0.9947	4.10	4.82	218.00	4.502	2.140	443.03	0.9892	5.84	36.94
**ZnAl-LDH-C**	130.00	138.86	0.009194	86.91	0.9968	2.59	6.81	187.35	4.192	1.471	225.29	0.9917	4.16	44.12
**ZnAl-LDH-S**	98.24	111.65	0.006805	65.96	0.9956	2.25	13.65	160.27	3.237	0.8315	127.37	0.9915	3.13	63.15
**ZnAl-LDH-CS**	122.64	134.86	0.007489	74.01	0.9968	2.39	9.97	189.90	3.108	1.121	170.13	0.9927	3.62	54.85

## Data Availability

The original contributions presented in this study are included in the article/Supplementary Materials. Further inquiries can be directed to the corresponding authors.

## Supplementary Materials

The following supporting information can be downloaded at: https://www.mdpi.com/article/10.3390/polym18182248/s1, Table S1. Representative water contact angle images and measured contact angles of ZnAl-LDH, starch–ZnAl-LDH, calcined ZnAl-LDH and calcined starch–ZnAl-LDH membranes.
