# Supplementary material for: Starch–ZnAl Layered Double-Hydroxide Nanocomposites and PVDF Membrane Nanofillers for the Sustainable Recovery of Dye-Contaminated Water
Source: Polymers (Basel). 2026 Sep 15;18(18):2248. doi: 10.3390/polym18182248 (PMC13611427; doi:10.3390/polym18182248)
Supplement: Supplementary file 1 [file polymers-18-02248-s001.zip › polymers-4436908-supplementary.pdf]

Supplementary Materials

Membrane type	Contact angle captured image	Contact angle
ZnAl-LDH Membrane (ZAL)	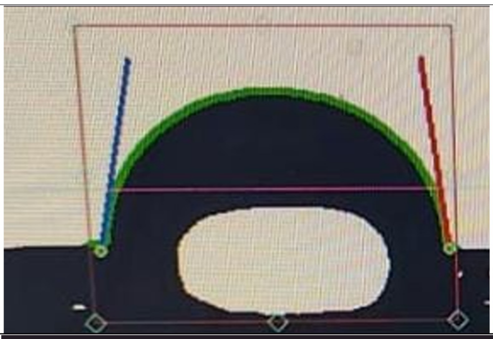	**79.8°**
Starch-ZnAl-LDH Membrane (S-ZAL)	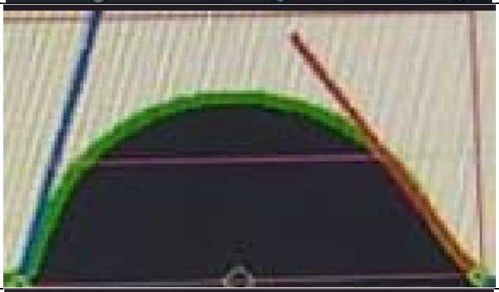	**67.4°**
Calcined ZnAl-LDH Membrane (C-ZAL)	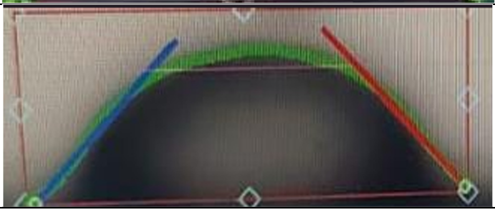	**44.3°**
Calcined Starch-ZnAl-LDH Membrane (SC-ZAL)	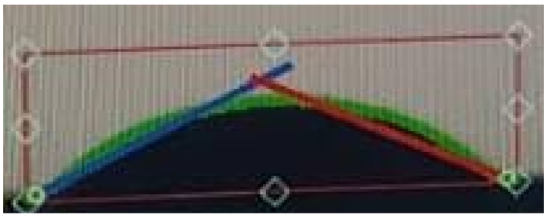	**25.8°**

Table S1. Representative water contact angle images and measured contact angles of ZnAl-LDH (ZAL), starch–ZnAl-LDH (S-ZAL), calcined ZnAl-LDH (C-ZAL), and calcined starch–ZnAl-LDH (SC-ZAL) membranes.
